# Protective effect of total flavonoids from *Ixeris Sonchifolia* on herpes simplex virus keratitis in mice

**DOI:** 10.1186/s12906-020-02911-w

**Published:** 2020-04-15

**Authors:** Yong-Qiang Wang, Li Cai, Nan Zhang, Jing Zhang, Hai-Hong Wang, Wei Zhu

**Affiliations:** 1 Department of Dermatology, Xuanwu Hospital, Capital Medical University, Beijing, 100053 China; 2 Department of Dermatology, Hebei General Hospital, Shijiazhuang, 050051 Hebei China; 3 Department of Dermatology, Baoding First Central Hospital, Baoding, 071000 Hebei China

**Keywords:** *Ixeris Sonchifolia* (Bae.) Hance, Herpes simplex keratitis, Total flavonoids, Anti-inflammation, IL-2

## Abstract

**Background:**

To investigate the protective effect of *Ixeris Sonchifolia* (Bae.) Hance (ISH) extract on herpes simplex virus keratitis (HSK) in mice.

**Methods:**

A mouse model of HSK was established by inoculating 60 mice (60 right eyes) with herpes simplex virus type 1 (HSV-1) by corneal scratch. The other 15 mice as blank control only received corneal scratch but without HSV-1. From the 2nd day after the successful modeling, the experimental group was fed with ISH total flavonoids (50, 100 and 200 mg/kg) orally, twice a day for 14 days. The model group and control group were given the same amount of normal saline. The pathological changes of cornea were observed once a day by slit lamp microscopy combined with fluorescein staining. The corneal histopathological examination, the survival status and the serum levels of interleukin-2 (IL-2), IL-4 and interferon-gama (INF-γ) were performed at the end of the experiment.

**Results:**

The result showed that ISH could significantly improve the corneal lesion degree, increase mice survival rate, and markedly increase the levels of IL-2 and INF-γ, reduce the levels of IL-4 in serum of mice.

**Conclusions:**

ISH could increase the anti-virus ability, promote the healing of corneal inflammation and alleviate the pathological damage of cornea, which suggested that ISH has a potential and valuable therapeutic effect on the HSK.

## Background

Viral keratitis is an inflammation of cornea caused by the infection of viral pathogens, which is one of the most serious blinding diseases in the world [1]. Viral keratitis is clinically divided into herpes simplex keratitis, varicella keratitis, herpes zoster keratitis and so on. Epidemiological investigation shows that herpes simplex virus infection is the most common form of viral keratitis. Herpes simplex keratitis (HSK) is a corneal infection caused by herpes simplex virus (HSV), it is a serious worldwide blinding eye disease, and its incidence and blindness rate occupy the first place in corneal diseases [2]. There are two kinds of viral keratitis: primary and recurrent. About 10% of the primary patients have clinical symptoms, and most of them are recurrent infections in clinic. Recurrent infections are mainly manifested as dendritic, mapped and discoid keratitis, often with a long course of illness and easy recurrence of symptoms, which ultimately affects visual function and the appearance of the eye [3]. The characteristics of this disease are difficult to cure, easy to recur and long course of disease. Its clinical manifestations are corneal trauma, and fever. The patients usually show symptoms of pain, redness, tears and photophobia. The patients have irritation symptoms and visual impairment. In severe cases, it can occur with pale, sparse pyorrhea and severe pain [4].

At present, drug treatment is still the main cure method for HSK. In the past, the drugs commonly used antiviral eye drops, which is mainly the synthetic nucleoxirus drugs [5, 6]. However, due to the drug resistance, it is not good effect in the clinical application, especially for the repeated HSK [7]. Thus, there is no definite effective drug available to intervene the recurrence of HSK. Exploring the pathogenesis of HSK and seeking alternative therapeutic drugs or methods have become a hot spot in clinical research [8]. Recently, many ophthalmologists try to find effective anti-HSV methods and drugs from traditional Chinese medicine. In order to develop new drugs with low toxicity, high efficiency and not resistant to drug resistance, the research of traditional Chinese medicine (TCM) is an important way that cannot be ignored.

Kudiezi is the whole grass or root of the Compositae plant *Ixeris Sonchifolia* (Bae.) Hance. It mainly used to treat coronary heart disease in clinic [9]. Modern pharmalogical experiment showed that Kudiezi has many biological activities such as anti-oxidant, anti-inflammation, anti-tumor and regulate the immune functions [10]. In celluar in vitro experiment, Karki et al. reported that the component of ISH have a potent anti-inflammatory activity in lipopolysaccharide-stimulated RAW 264.7 cells [11]. The study of Yang et al. showed that ISH have a strong anti-oxidant activity in vitro [12]. In addition, there also some studies showed that ISH provide a neuroprotective effects against ischemia-induced cellular injury in vitro [13], and inhibition the cell proliferation in HepG2 human hepatocellular carcinoma cells [14]. More importantly, in animal model experiment, ISH showed a potent anti-allergic effects in mice model [15], anti-tumors in non-small cell lung cancer in animals [16], and anti-human hepatocellular carcinoma in mice model [14]. Furthermore, the constitutes study of the ISH showed that the main bioactivity component mainly contains flavonoid [17], sesquiterpene lactone [18, 19], sesquiterpene lactone glucoside [20], triterpenoidal saponin [21–23] and etc.. In above, based on the previous research or theory, we wondered that whether ISH have the antiviral effects?

In the study, we used the HSV-1 infected mice model to study the effect and potential mechanisms of ISH total flavonoids on HSK, which could be provide the theoretical basis for HSK therapy in clinic.

## Methods

### Materials and reagents

Mouse interferon-gama (INF-γ, Batch No 978941030), interleukin-2 (IL-2, Batch No 948941121) and interleukin-6 (IL-4, Batch No 948941123) were purchased from Shenzhen Jingmei Bioengineering Co., Ltd. (Shenzhen, China). 1% Sodium fluorescein eye drops obtained from the preparation room of Xuanwu Hospital, Capital Medical University (Beijing, China).

Total flavonoids from *Ixeris Sonchifolia* (batch number: 20171121), a yellow brown dry powder, containing the total flavonoids of 98.7%, was purchased from Beijing Traditional Chinese Medicine Factory (Beijing, China). The total flavonoids were obtained according to the reference of Chen et al. reported [24], and the products was also identified by Professor Wei Zhu from Capital Medical University according to the reference [24].

### Virus information

Standard herpes simplex virus type I (HSV-1) SM44 strain originated from Virus Seed Room of Virus Prevention and Control Institute, Chinese Center for Disease Control and Prevention. The virus was passed on for three generations in human embryonic renal epithelial cells (HEK293T) before use to be activated. The cytopathic changes were observed, and the virus titer TCID_50_ (Tissue culture infective dose of half cytopathy) was calculated. The TCID_50_ of the virus is 10^–9.31^/0.l mL, that is, 0.l mL per hole of the virus with titer of 10^–9.31^ can cause 50% cells to have obvious pathological changes. The HEK293T cell line was obtained from Cell Bank of Chinese Academy of Sciences (China), and culture in the medium of DMEM (Dulbecco's Modified Eagle Medium, Gibco) + 10% FBS (fetal bovine serum, Gibco).

### Animals

Seventy-five adult male BALB/c mice (body weight 20 ± 2 g) aged 6 weeks were provided by the animal experimental center of Capital Medical University (Beijing, China). All the mice were reared in SPF grade environment with the temperature and humidity of 24 ± 1 °C and 50% ± 5% under the 12 h day/night cycle in the animal experimental center of Capital Medical University. Six mice were raised in one polyacrylic cage with free access to food and water, the mice were quarantined for 1 week before the experiment use. All the mice were received humane care according to the terms of National Institutes of Health Guidelines of the USA (National Research Council of USA, 1996) and the University ethical regulations of Capital Medical University.

### HSK animal model establish

The HSK animal model establish process as follows: First, mice were anaesthetized by isoflurane inhalation. Use the sterile BD needle to mark out a “#” font in the epithelial layer of the right eye cornea under the microscope. For the model mice, dripped 5 μL of the DMEM containing the virus with TCID_50_ of 10^–9.31^/0.l mL onto the surface of the right eye cornea, and massaged the eyelids of the mice gently for 2 min. While for the control group mice, dripped 5 μL of DMEM without virus onto the surface of the right eye cornea, and massaged the eyelids of the mice gently for 2 min.

### Experimental design

**Fig. 1:**
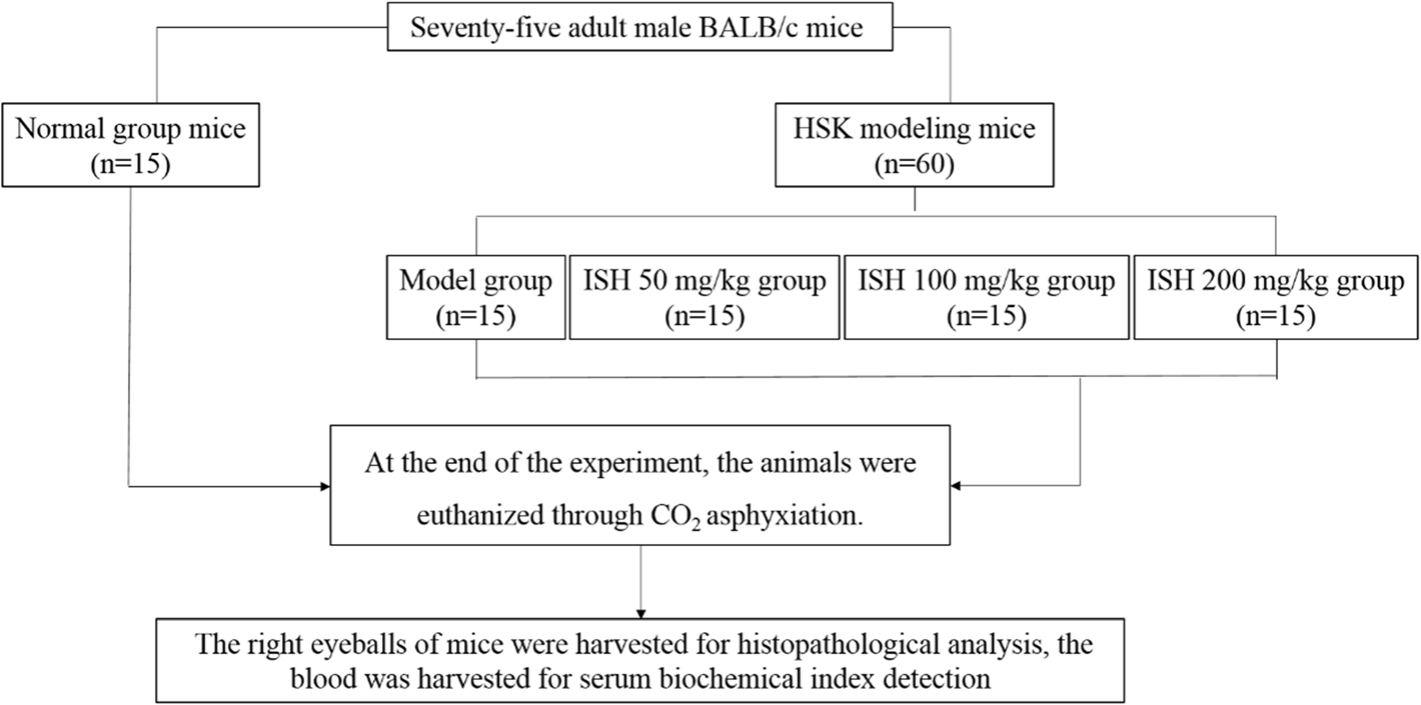
The graphic of the experimental design

The completely animal experiment were performed at animal experimental center of Capital Medical University, and the experimental protocol was approved by animal care and use committee of Capital Medical University. Sixty modeling mice were randomly divided into 4 groups, 15 mice per group (details see Fig. 1): the model group, ISH 50, 100 and 200 mg/kg groups. The mice were oral administrated with the related dosage of ISH, twice a day for 14 days. The normal and model group were oral administrated with saline for 14 days. At the end of the experiment, the animals were euthanized through CO_2_ asphyxiation using slow displacement of chamber air with compressed CO_2_ (25%/min).

### Criteria for corneal lesion degree

The condition of corneal lesions was observed through slit lamp microscopy every day. Corneal epithelial lesions were divided into 0–4 degrees according to the corneal area [25–27]. 0 degrees: no keratopathy, no staining and opacity; 1 degree: corneal epithelial lesions accounted for less than 25% of the whole cornea; 2 degrees: corneal epithelial lesions accounted for 25–50% of the whole cornea; 3 degrees: corneal epithelial lesions accounted for 51–75% of the whole cornea; 4 degrees: corneal epithelial lesions accounted for more than 75% of the whole cornea.

In addition, the criteria of HSK cure as follows: the irritation symptoms and ciliary congestion disappeared, corneal epithelium was repaired, fluorescein staining was negative, corneal edema infiltrated and subsided, aqueous flash was negative, corneal epithelium and lacrimal virus were negative.

### Histopathological analysis

After drug administration, the right eyeballs of mice were harvested, and fixed with 10% formaldehyde for overnight. Then the fixed samples embedded in paraffin, and then cut into 4 μm thickness sections by Leicia 2128 tissue microtome. The eye sections then stained with hematoxylin/eosin (H&E) according to the terms of the routine histopathological examination. The final stained sections were photographed under a light microscope (BX-50 Olympus) at 200× magnification.

### Detection of INF-γ, IL-2 and IL-4 expression in serum of mice by ELISA

The expressions of INF-γ, IL-2 and IL-4 in serum of mice in each group were detected by ELISA kit according to the manufactory instructions of Shenzhen Jingmei Bioengineering Co., Ltd., (Shenzhen, China).

### Statistical analysis

The values were showed as mean ± SD. All statistical comparisons were calculated by means of a one-way ANOVA test followed by Dunett’s t-test with SPSS19.0 statistical software. *P* < 0.05 and < 0.01 were regarded as statistically significant.

## Results

### HSV-1 infect detection

**Fig. 2:**
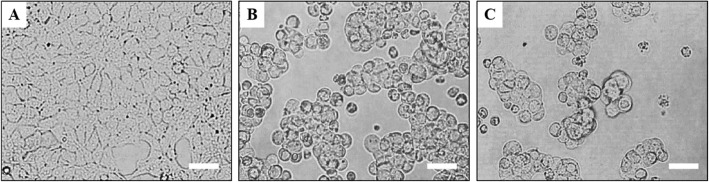
HEK293T cell morphology before and after HSV-1 infection

In order to detect if the animal model was successfully established, HEK293T normal cells were used to the virus infection detection object. The detection method as follows: from the third day after HSV-1 infection, the tear film on the corneal surface of the right eye was wiped 8 times with a wet cotton swab (dipped with DMEM) every day, then placed the wet cotton swab into a EP tube (filled with 1 mL DMEM culture medium) for blowing 6 times. Take 200 μL corneal wipe solution to transplant into the culture flask of HEK293T cells, then cultured in 37 °C, 5% CO_2_ incubator for 24 h and 36 h respectively, to observe whether the HEK293T cells showed cytopathological changes (CPE). If HEK293T cells become large, round, grape-shape aggregation and float on the cell culture medium, it means the cell is CPE positive (Fig. 2), there was virus replication in the wipe fluid, and it suggesting that the model was successful. While from the whole experiment, we found that the morbidity of the mice in drug treatment group and model group was 100% virus infection.

### Clinical characteristics of HSV-1 infected mice

**Fig. 3:**
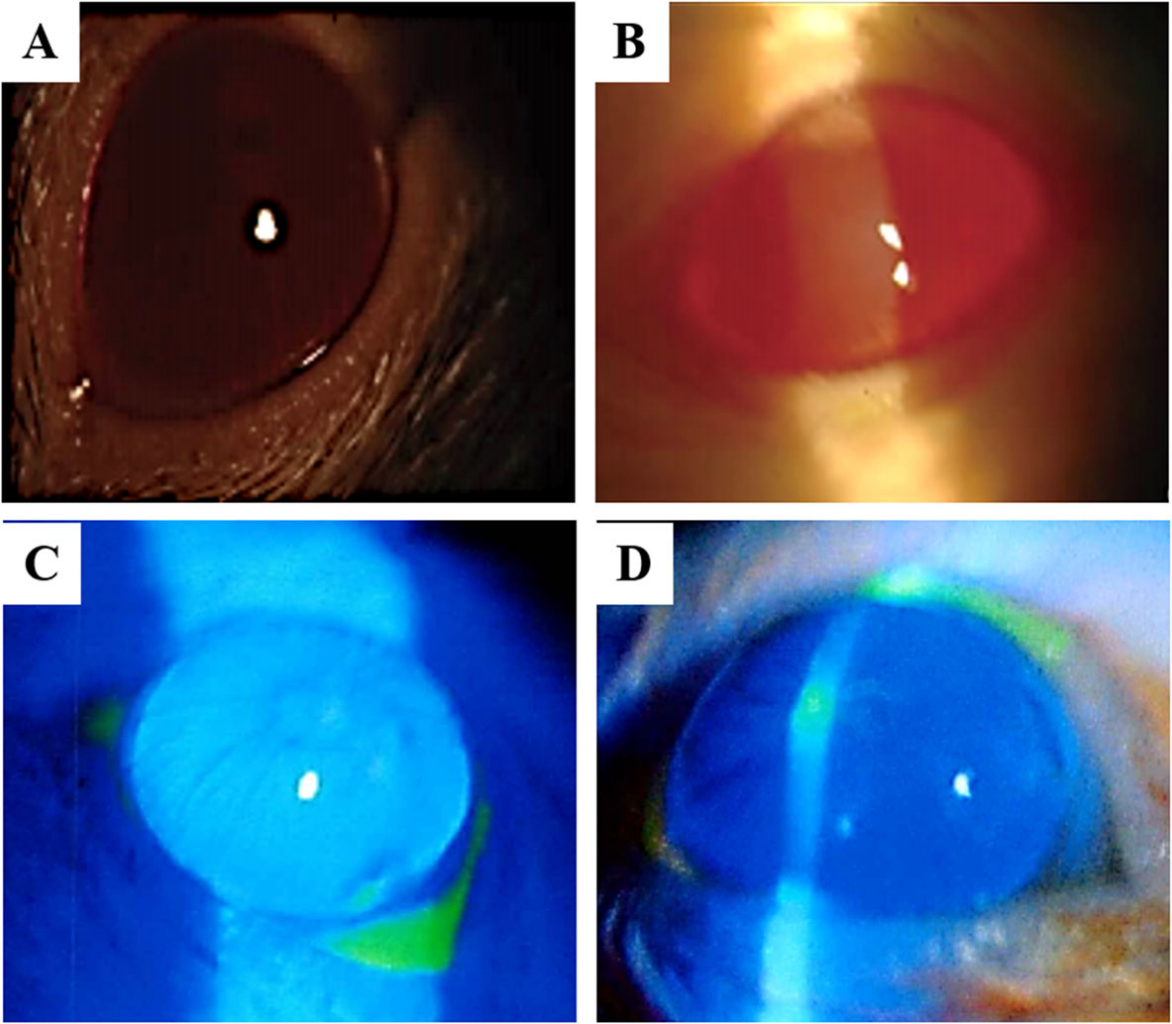
Corneal changes of mice before and after HSV-l infection (3 days). (A/B) Corneal morphology of normal (**a**) and model (**b**) mice group under the slit-lamp microscope. (C/D) Corneal morphology of normal (**c**) and model (**d**) mice group after fluorescein sodium staining in the right eye of mice

In healthy control group, the corneal epithelial scratches of the mice was healed, the corneal showed smooth, and no abnormal changes were observed after 1 day of the corneal scratches (Fig. 3a, c). In model group, the corneal of the mice showed punctate epithelioid and persistent epithelial defect after 3 days of HSV-1 inoculation (Fig. 3b, d); neovascularization and corneal ulcer were seen in the limbus of cornea at 6 days of HSV-1 inoculation; corneal ulcer deepened, corneal edema and neovascularization grew into the central cornea at 8 days; corneal ulcer healed partially at 14 days, among them 2 mice showed a corneal ulcer perforation in right eyes, cornea edema with gray-white water, and neovascularization that extends beyond the central cornea to cover the entire cornea. In drug treatment groups, dendritic epithelioid appeared at 3 days after HSV-1 inoculation; neovascularization and corneal edema appeared at 7 days, corneal ulcer appeared in some mice; corneal ulcer basically stationary, corneal edema was not obvious, and corneal central neovascularization decreased compared with model group at 14 days.

### Pathological scores of mice corneal and mice morbidity

**Fig. 4:**
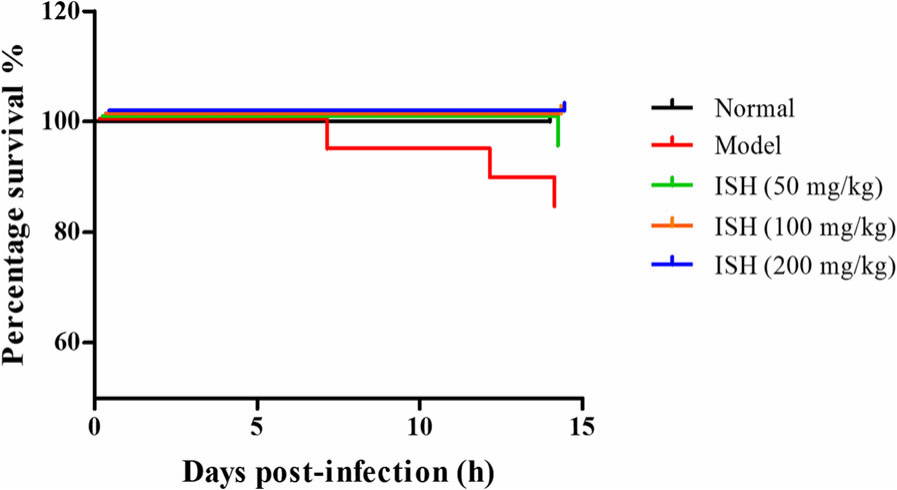
The survival rate of mice in the whole experiment (*n* = 15). ISH, *Ixeris Sonchifolia* (Bae.) Hance

Next, we evaluated the degrees of the corneal lesion in each mice of each group. In healthy control group, the cornea of the mice were all normal and smooth, and all the mice were lived in the whole experiment. In model group, 1 mice was dead in the 7th day and 2 mice were dead in the 14th day (Fig. 4). There was 1 mice recovered to normal cornea thoroughly at 14 days (Table 1). In drug treated groups, the corneal lesion were gradually recovered to normal with the treatment days, especially in the high dose of the drug treatment group (Table 1). In 50 mg/kg group, 1 mice was dead in 14th day (Fig. 4), 33.3% mice were thoroughly recovered, and only 26.6% mice were in the 2 degree at 14 days treatment (Table 1). In 100 mg/kg group, all the mice were survived, 46.4% mice were thoroughly recovered, and only 20% mice were in the 2 degree at 14 days treatment (Table 1). In 200 mg/kg group, all the mice were survived, 60% mice were thoroughly recovered, and only 6.6% mice were in the 2 degree at 14 days treatment (Table 1). The details of the corneal lesion degrees in each group were showed in Table 1.

**Table 1:** The scores of corneal lesion in each group during the whole experiment

Group	Dose	n	CLD before ISH treatment				
			0	1	2	3	4
Normal	–	15	15	0	0	0	0
Model	–	15	0	3	5	5	2
ISH-low	50 mg/kg	15	0	2	6	5	2
ISH-medium	100 mg/kg	15	0	2	5	6	2
ISH-high	200 mg/kg	15	0	1	5	6	3
Group	Dose	n	CLD after 3 days of ISH treatment				
			0	1	2	3	4
Normal	–	15	15	0	0	0	0
Model	–	15	0	3	6	4	2
ISH-low	50 mg/kg	15	0	3	7	4	1
ISH-medium	100 mg/kg	15	0	4	6	4	1
ISH-high	200 mg/kg	15	2	3	5	4	1
Group	Dose	n	CLD after 7 days of ISH treatment				
			0	1	2	3	4
Normal	–	15	15	0	0	0	0
Model	–	14	0	4	5	4	1
ISH-low	50 mg/kg	15	2	4	6	3	0
ISH-medium	100 mg/kg	15	3	5	4	3	0
ISH-high	200 mg/kg	15	6	4	4	1	0
Group	Dose	n	CLD after 14 days of ISH treatment				
			0	1	2	3	4
Normal	–	15	15	0	0	0	0
Model	–	12	1	4	4	3	0
ISH-low	50 mg/kg	14	5	5	4	0	0
ISH-medium	100 mg/kg	15	7	5	3	0	0
ISH-high	200 mg/kg	15	9	5	1	0	0

CLD, Corneal Lesion Degree; ISH, *Ixeris Sonchifolia* (Bae.) Hance

### Histophaological detection

**Fig. 5:**
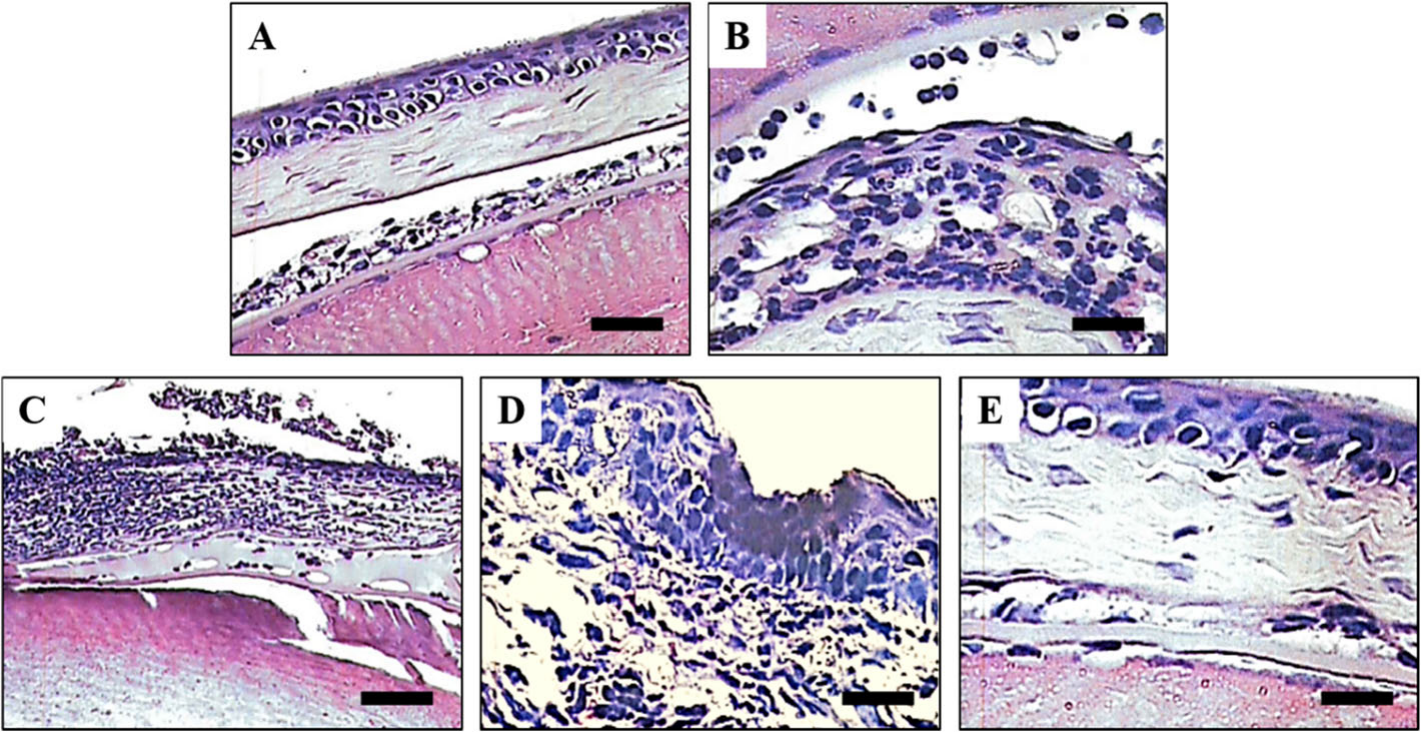
Histopathological changes of mice corneal. **a.** Normal group, **b.** Model group, **c**. ISH 50 mg/kg group, **d**. ISH 100 mg/kg group, E. ISH 200 mg/kg group. ISH, *Ixeris Sonchifolia* (Bae.) ance. Scale bar, 50 μm

The histophaological detection by H&E staining was showed in Fig. 5. In control group, the corneal epithelium of mice was intact without edema and inflammation cells (Fig. 5a). In the model group, the corneal epithelium showed extensive erosion, corneal stroma edema, obvious inflammation in the stroma, and large numbers of inflammatory cells infiltration (Fig. 5b). In the drug treatment groups, the corneal edema and the stroma inflammation were obviously decrease compared with the model group, which showed a dose-dependent manner (Fig. 5c, d, e).

### Effect of ISH on serum levels of INF-*γ*, IL-2 and IL-4

The serum levels of inflammation factors were detected and showed in Table 2. After HSV-1 infection, the serum levels of INF-*γ* and IL-2 were significantly decreased in the model group, while IL-4 was markedly increased (*P* < 0.01, Table 2), compared with the control group. After 14 days drug treatment, the levels of INF-*γ* and IL-2 were obviously increase in each ISH treated group, compared with the model group (*P* < 0.05, *P* < 0.01, Table 2). With the consistent effect, the levels of IL-4 was significantly decrease, compared with the model group (*P* < 0.05, *P* < 0.01, Table 1).

**Table 2:** The levels of INF-γ, IL-2 and IL-4 in mice serum

Group	Dose	INF-γ (pg/mL)	IL-2 (ng/L)	IL-4 (ng/L)
Normal	–	73.55 ± 3.36	16.57 ± 1.35	26.30 ± 2.41
Model	–	25.16 ± 2.57^##^	8.34 ± 1.01^##^	42.43 ± 2.08^##^
ISH-low	50 mg/kg	39.98 ± 4.04^*^	9.21 ± 1.16^*^	33.05 ± 1.17^*^
ISH-medium	100 mg/kg	60.91 ± 5.37^**^	10.26 ± 1.73^*^	29.13 ± 1.79^**^
ISH-high	200 mg/kg	70.35 ± 4.17^**^	16.74 ± 1.28^**^	25.11 ± 2.02^**^

Data are expressed as mean ± SD for each group. ^##^ P < 0.01 vs normal group; ^*^P < 0.05, ^**^P < 0.01 vs model normal group. ISH, *Ixeris Sonchifolia* (Bae.) Hance

## Discussion

HSK belongs to the category of “Juxing Barrier” in TCM. Its etiology and pathogenesis are mainly due to heat accumulation or Yin deficiency, wind-heat toxin, stagnation and persistence of fever, and finally lead to the eye disease [28, 29]. Currently, the clinical treatment of HSK is still a very difficult problem. Although there are many therapies, the specific mechanism of HSK is still unclear, so the therapeutic effect cannot be effectively controlled [30]. In current, western medicine treatment is mainly the antiviral drugs, which is focus on the inhibiting amplification of viral DNA to achieve the improving patient’s clinical symptoms. Among them, Acyclovir is the most widely used, but it has a greater impact on the body’s immune system, and is prone to drug resistance, not suitable for long-term treatment. The existing therapies cannot completely cure HSK, they can only alleviate symptoms and maintain the latent state of virus [31, 32].

Recently, many ophthalmologists try to find effective anti-HSV methods and drugs from traditional Chinese medicine. In order to develop new drugs with low toxicity, high efficiency and no drug resistance, the research of TCM has become a hotspot for disease treatment [33]. The antiviral mechanism of TCM preparations, on the one hand, may be related to the direct elimination of viruses or induction of interferons, reduction or blocking of the translation of viral RNA, on the other hand, it may be related to the adjustment of the immune system and the enhancement of immune function, while the latter seems more important [34, 35]. TCM has its advantages in adjusting immune function, strengthening the body and eliminating pathogens. In addition, TCM has a light stimulating effect, toxicity and side effects, which is superior to Western medicine. In our study, the ISH treatment significantly alleviate the damage of HSV induced mice. The mice mortality in the untreated model mice was 20%, in ISH 50 mg/kg group is 6.7%, while in 100 and 200 mg/kg ISH treated group is 0%, which implied that ISH may be a potential effective drug for HSV infection in future clinical therapy.

Corneal fluorescein staining is a commonly used method in clinical examination of corneal diseases [36]. Sodium fluorescein, as a staining agent, is stained when corneal lesions occur [37]. When HSV infects cornea, the corneal lesion usually only extends to the superficial layer. Under slit lamp microscopy, the epithelial defect of corneal ulcer can be clearly seen to be dark green stained by fluorescein, while the periphery of the ulcer is surrounded by light green infiltration margin [38, 39]. In our study, the corneal changes of mice under the slit-lamp microscope was effectively guided the success of mice modeling establish.

In addition, IL-2 and IFN-γ, the necessary inflammatory regulators, belongs to Th1 cytokines, which can enhance the cellular immune function, mediate the cellular immune response, and play an active role in antiviral and bacterial infection. IL-4 is the key factor to promote Th0 cells to differentiate into Th2 cells, but it, together with IL-13, inhibits the differentiation and function of Th1 cells. It is belongs to Th2 cytokines, which mainly participate in the acute hypersensitivity reaction, have auxiliary effect on all the antibodies synthesized by B-lymphocytes, have inhibitory effect on cell immunity, and mediate the immune response of the fluid. IL-4 can stimulate the proliferation of B cells, promote the expression of MHC II antigen and CD40, enhance the antigen ability of B cells, and promote the production of IgE in B cells. IFN-γ can inhibit the above biological functions of IL-4, and IL-4 can also inhibit the transcription of IFN-γ mRNA [40]. Th1/Th2 cell balance is an important mechanism to maintain physiological stability. In our study, the result showed that the mice treated with ISH were significantly improved the serum levels of IL-2, INF-γ and IL-4, which exhibited that the recovery of the balance of Th1/Th2 in the immnoregulation system of mice.

## Conclusion

In conclusion, ISH could increase the mice anti-virus ability, promote the healing of corneal inflammation, and alleviate the pathological damage of cornea, which indicated that ISH may be a potent and valuable agent for the therapy of HSK in futrue clinic.

## Data Availability

The datasets used and/or analyzed during the current study are available from the corresponding author on reasonable request.

## Abbreviations

ISH: *Ixeris Sonchifolia* (Bae.) Hance
HSK: Herpes simplex virus keratitis
HSV-1: Herpes simplex virus-1
H&E: Hematoxylin and eosin
INF-γ: Interferon-gama
IL-2: Interleukin-2
IL-4: Interleukin-4

## References

[1] Lobo AM, Agelidis AM, Shukla D (2019). Pathogenesis of herpes simplex keratitis: the host cell response and ocular surface sequelae to infection and inflammation. Ocul Surf.

[2] Roozbahani M, Hammersmith KM (2018). Management of herpes simplex virus epithelial keratitis. Curr Opin Ophthalmol.

[3] Rajasagi NK, Rouse BT (2018). Application of our understanding of pathogenesis of herpetic stromal keratitis for novel therapy. Microbes Infect.

[4] Azher TN, Yin XT, Tajfirouz D, Huang AJ, Stuart PM (2017). Herpes simplex keratitis: challenges in diagnosis and clinical management. Clin Ophthalmol.

[5] Bhatt UK, Abdul Karim MN, Prydal JI, Maharajan SV, Fares U (2016). Oral antivirals for preventing recurrent herpes simplex keratitis in people with corneal grafts. Cochrane Database Syst Rev.

[6] Solanki S, Rathi M, Khanduja S, Dhull CS, Sachdeva S, Phogat J (2015). Recent trends: medical management of infectious keratitis. Oman J Ophthalmol.

[7] Tsatsos M, MacGregor C, Athanasiadis I, Moschos MM, Hossain P, Anderson D (2016). Herpes simplex virus keratitis: an update of the pathogenesis and current treatment with oral and topical antiviral agents. Clin Exp Ophthalmol.

[8] Rolinski J, Hus I (2014). Immunological aspects of acute and recurrent herpes simplex keratitis. J Immunol Res.

[9] Gao SS, Cui RZ, Xie YM, Liao X, Gao XY, Wang JD (2017). Systematic review of Kudiezi injection drug safety. Zhongguo Zhong yao za zhi = Zhongguo zhongyao zazhi = Chin J Chin Mater Med.

[10] Liu X, Jin X, Chen B, Liu X, Liang X, Fang X, Wu H, Fu X, Zheng H, Ding X (2018). Effects of Kudiezi injection on serum inflammatory biomarkers in patients with acute cerebral infarction. Dis Markers.

[11] Karki S, Park HJ, Nugroho A, Kim EJ, Jung HA, Choi JS (2015). Quantification of major compounds from Ixeris dentata, Ixeris dentata Var. albiflora, and Ixeris sonchifolia and their comparative anti-inflammatory activity in lipopolysaccharide-stimulated RAW 264.7 cells. J Med Food.

[12] Yang L, Sun G, Guo Y, Hou Z, Chen S (2016). Holistic evaluation of quality consistency of Ixeris sonchifolia (Bunge) Hance Injectables by quantitative fingerprinting in combination with antioxidant activity and Chemometric methods. PLoS One.

[13] Zhang YC, Gan FF, Shelar SB, Ng KY, Chew EH (2013). Antioxidant and Nrf2 inducing activities of luteolin, a flavonoid constituent in Ixeris sonchifolia Hance, provide neuroprotective effects against ischemia-induced cellular injury. Food Chem Toxicol : Int J Published Br Ind Biol Res Assoc.

[14] Yee SB, Lee JH, Chung HY, Im KS, Bae SJ, Choi JS, Kim ND (2003). Inhibitory effects of luteolin isolated from Ixeris sonchifolia Hance on the proliferation of HepG2 human hepatocellular carcinoma cells. Arch Pharm Res.

[15] Trinh HT, Bae EA, Hyun YJ, Jang YA, Yun HK, Hong SS, Kim DH (2010). Anti-allergic effects of fermented Ixeris sonchifolia and its constituent in mice. J Microbiol Biotechnol.

[16] Zhang YC, Zhou L, Ng KY (2009). Sesquiterpene lactones from Ixeris sonchifolia Hance and their cytotoxicities on A549 human non-small cell lung cancer cells. J Asian Nat Prod Res.

[17] Lu J, Feng X, Sun Q, Lu H, Manabe M, Sugahara K, Ma D, Sagara Y, Kodama H (2002). Effect of six flavonoid compounds from Ixeris sonchifolia on stimulus-induced superoxide generation and tyrosyl phosphorylation in human neutrophils. Clin Chimica Acta; Int J Clin Chem.

[18] Song SJ, Zhou LY, Li LZ, Gao PY, Jia WW, Peng Y (2011). Two new sesquiterpene lactones from Ixeris sonchifolia. Nat Prod Commun.

[19] Zhang WZ, Li XL, Shi LG, Wang JL, Zhao M, Zhao DF, Zhang SJ (2008). Sesquiterpene lactones from Ixeris sonchifolia (Bge.) Hance II. J Asian Nat Prod Res.

[20] Feng XZ, Xu SX, Dong M (2001). A new sesquiterpene lactone glucoside from Ixeris sonchifolia. J Asian Nat Prod Res.

[21] Feng XZ, Dong M, Gao ZJ, Xu SX (2003). Three new triterpenoid saponins from Ixeris sonchifolia and their cytotoxic activity. Planta Med.

[22] Suh J, Jo Y, Kim ND, Bae SJ, Jung JH, Im KS (2002). Cytotoxic constituents of the leaves of Ixeris sonchifolia. Arch Pharm Res.

[23] Feng XZ, Dong M, Xu SX (2001). A new triterpenoidal saponin from Ixeris sonchifolia and its cytotoxic activity. Die Pharmazie.

[24] Chen Y, Jiang X, Luo H, Jiang R (2011). Study on the extraction process of total flavonoids from Ixeris sonchifolia (Bge.) Hance. Specl Wild Econ Anim Plant Res.

[25] Trousdale MD, Barlow WE, McGuigan LJ (1989). Assessment of diclofenac on herpes keratitis in rabbit eyes. Arch Ophthalmol.

[26] Kaufman HE, Varnell ED, Thompson HW (1999). Latanoprost increases the severity and recurrence of herpetic keratitis in the rabbit. Am J Ophthalmol.

[27] Asbell PA (2000). Valacyclovir for the prevention of recurrent herpes simplex virus eye disease after excimer laser photokeratectomy. Trans Am Ophthalmol Soc.

[28] Rowe AM, St Leger AJ, Jeon S, Dhaliwal DK, Knickelbein JE, Hendricks RL (2013). Herpes keratitis. Prog Retin Eye Res.

[29] Harris KD. Herpes Simplex Virus Keratitis. Home Healthc Now. 2019;37(5):281–4. 10.1097/NHH.0000000000000791 31483360

[30] Jahanban-Esfahlan R, Seidi K, Majidinia M, Karimian A, Yousefi B, Nabavi SM, Astani A, Berindan-Neagoe I, Gulei D, Fallarino F (2019). Toll-like receptors as novel therapeutic targets for herpes simplex virus infection. Rev Med Virol.

[31] Serna-Ojeda JC (2019). Management of Herpes Simplex Virus Keratitis in the pediatric population. Pediatr Infect Dis J.

[32] Abdelmassih Y, Dubrulle P, Sitbon C, El-Khoury S, Guindolet D, Doan S, Labetoulle M, Cochereau I, Gabison EE (2019). Therapeutic challenges and prognosis of Descemet's membrane endothelial Keratoplasty in herpes simplex eye disease. Cornea.

[33] Whitley R, Baines J. Clinical management of herpes simplex virus infections: past, present, and future. F1000Research. 2018;7. 10.12688/f1000research.16157.1. 10.12688/f1000research.16157.1 PMC6213787 30443341

[34] Tang R, Zhai Y, Dong L, Malla T, Hu K (2018). Immunization with dendritic cell-based DNA vaccine pRSC-NLDC145.gD-IL21 protects mice against herpes simplex virus keratitis. Immunotherapy.

[35] Kalogeropoulos D, Geka A, Malamos K, Kanari M, Kalogeropoulos C (2017). New therapeutic perceptions in a patient with complicated herpes simplex virus 1 keratitis: a case report and review of the literature. Am J Case Rep.

[36] Amparo F, Wang H, Yin J, Marmalidou A, Dana R (2017). Evaluating corneal fluorescein staining using a novel automated method. Invest Ophthalmol Vis Sci.

[37] Bandamwar KL, Papas EB, Garrett Q (2014). Fluorescein staining and physiological state of corneal epithelial cells. Cont Lens Anterior Eye.

[38] Mecum NE, Cyr D, Malon J, Demers D, Cao L, Meng ID (2019). Evaluation of corneal damage after lacrimal gland excision in male and female mice. Invest Ophthalmol Vis Sci.

[39] Woods J, Hutchings N, Srinivasan S, Jones L. Geographic distribution of corneal staining in symptomatic dry eye. Ocul Surf. 2019. 10.1016/j.jtos.2019.07.009 31352082

[40] Ellermann-Eriksen S (2005). Macrophages and cytokines in the early defence against herpes simplex virus. Virol J.

